# Supplementary material for: Met regulates endoderm migration in zebrafish
Source: bioRxiv. 2026 May 5:2026.05.01.722050. Preprint. [Version 1] doi: 10.64898/2026.05.01.722050 (PMC13174448; doi:10.64898/2026.05.01.722050)
Supplement: Supplement 5 — Supplemental Figure 1. metucm123 exhibits delayed endoderm convergence and reduced migration persistence. (A–A″) Representative images of endoderm labeled with Tg(sox17:GFP) in wild-type (A), heterozygous (A′), and homozygous mutant (A″) metucm123 embryos at 11.5 hpf. Endoderm is outlined (dashed line), and endoderm width is indicated (solid line). Dorsal views, anterior towards the top. Scale bar, 150 μm (B) Quantification of relative endoderm width (endoderm/embryo width) at 11.5 hpf from wild-type, heterozygous, and homozygous mutant metucm123 embryos at 11.5 hpf. Points represent individual embryos 28 color coded by batch. Open circles indicate batch means. Error bars indicate standard deviation. p values determined by one-way ANOVA. +/+ (n = 32), +/− (n = 68), −/− (n = 37), from four independent batches each. (C–E″) Time-lapse confocal imaging of endoderm labeled with Tg(sox17:GFP) in wild-type (C–C″), heterozygous (D–D″), and homozygous mutant (E–E″) metucm123 embryos starting at 8.5 hpf. Embryos are shown at the start (C, D, E) and end (C′, D′, E′) of each time-lapse along with migration tracks generated by cells throughout the time-lapse (C″, D″, E″). Lateral views, dorsal to the right. Scale bar, 100 μm. (F–H) Quantification of migration displacement (F), velocity (G), and persistence (H) from wild-type, heterozygous, and homozygous mutant metucm123 embryos. Points represent individual cell tracks, color colored by embryo. Open circles depict embryo means. Error bars indicate standard deviation. p values determined by one-way ANOVA. n.s., not significant. +/+ (987 cells from 3 embryos), +/− (2835 cells from 9 embryos), −/− (832 cells from 3 embryos). [file NIHPP2026.05.01.722050v1-supplement-5.pdf]

bioRxiv preprint doi: https://doi.org/10.64898/2026.05.01.722050; this version posted May 5, 2026. The copyright holder for this preprint (which was not certified by peer review) is the author/funder, who has granted bioRxiv a license to display the preprint in perpetuity. It is made available under aCC-BY 4.0 International license.

Supplemental Figure 1

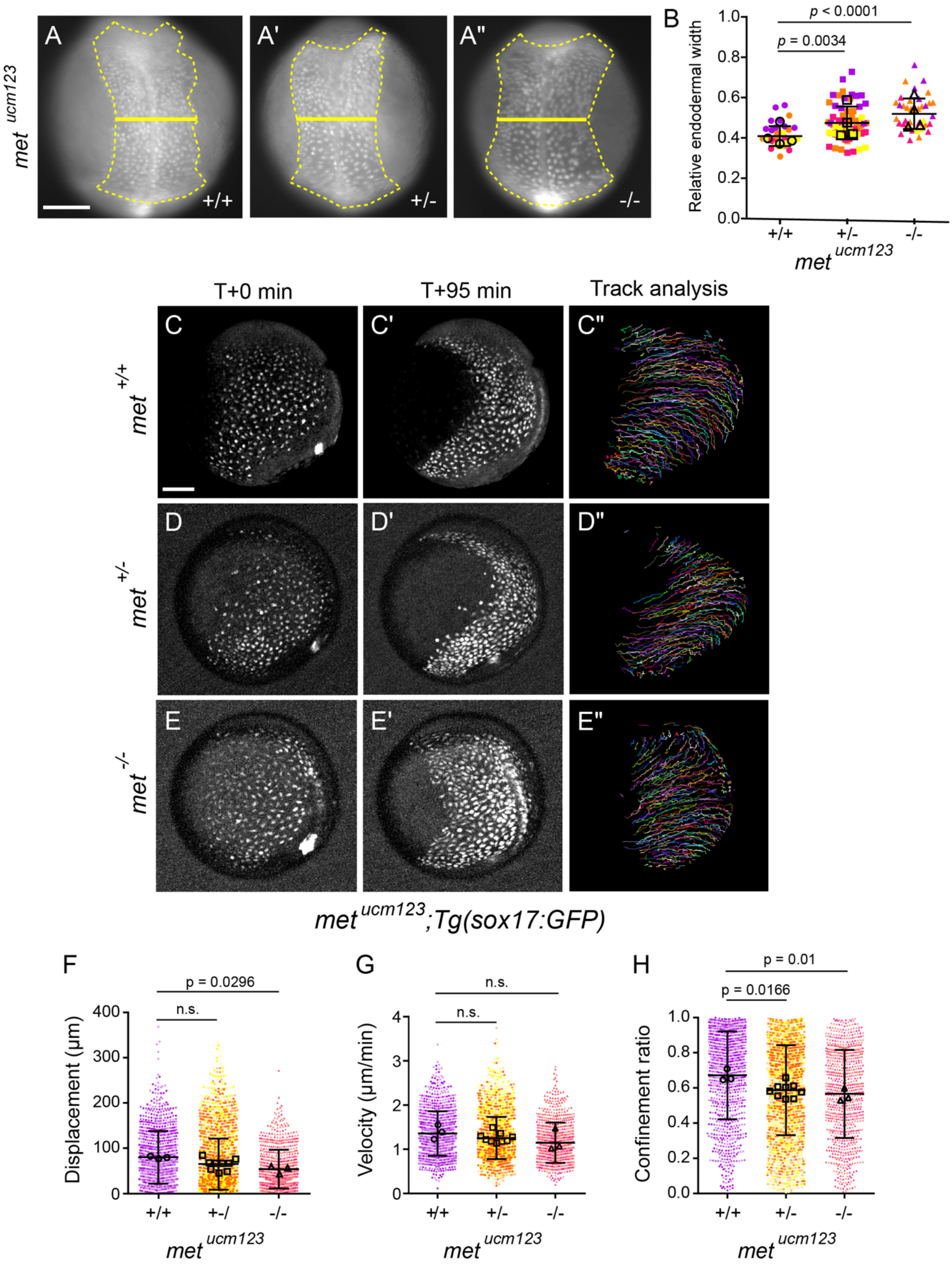

bioRxiv preprint doi: https://doi.org/10.64898/2026.05.01.722050; this version posted May 5, 2026. The copyright holder for this preprint (which was not certified by peer review) is the author/funder, who has granted bioRxiv a license to display the preprint in perpetuity. It is made available under aCC-BY 4.0 International license.

Reagent or resource	Source	Identifier
Zebrafish Strains		
Tg(*sox17*: GFP)	Chung and Stainier, 2008	ZDB-ALT-061228-2
hgfa^{fh528}	Isabella et al., 2020	ZDB-ALT-201203-10
met^{fh533}	Isabella et al., 2020	ZDB-ALT-201203-9
met^{ucm123}	This study	
met^{ucm124}	This study	
Chemicals		
SGX-523	MedChemExpress	HY-12019
INCB28060	MedChemExpress	HY-13404
Dimethyl sulfoxide (DMSO)	Sigma-Aldrich	D2650
Phosphate Buffered Saline (PBS)	ThermoFisher	BP399
Saline-Sodium Citrate (SSC)	ThermoFisher	BP1325
Methanol (MeOH)	ThermoFisher	A452
Glycerol	ThermoFisher	J61059
Agarose	VWR	0710
Biochemical Reagents		
Alt-R™ S.p. Cas9 Nuclease V3	IDT	1081058
*met*_tss_sgRNA: TAAATGATCCGTCGTCAATG	IDT	This study
*met*_e2_sgRNA: TCATTCAGAAGGGTTACACA	IDT	This study
hgfa morpholino (MO-*hgfa*)	Gene Tools	ZDB-MRPHLNO-100420-4
hgfb morpholino (MO-*hgfb*)	Gene Tools	ZDB-MRPHLNO-100420-3
met probes for HCR RNA-FISH	Molecular Instruments	XM_005162995.4
hgfa probes for HCR RNA-FISH	Molecular Instruments	XM_005164742.3
RNeasy Micro Kit	QIAGEN	74004
AllPrep DNA/RNA Micro Kit	QIAGEN	80204
ProtoScript II First Strand cDNA Synthesis Kit	NEB	E6560
PerfeCTa SYBR Green FastMix Low ROX	Quantabio	84073
GoTaq G2 Green Master Mix for PCR	Promega	M7823
Equipments and Tools		
QuantStudio 3 Real-Time PCR Systems	ThermoFisher	A28567
31G ultra-fine needle	BD Biosciences	328418
Glass-bottom 35mm Petri Dish	MATTEK	P35G-1.5-14-C
Software and Algorithms		
Prism	GraphPad	https://www.graphpad.com/
Fiji	ImageJ	https://imagej.net/
TrackMate	Tinevez et al., 2017	PMID: 27713081
